# Bridging health technology assessment (HTA) with multicriteria decision analyses (MCDA): field testing of the EVIDEM framework for coverage decisions by a public payer in Canada

**DOI:** 10.1186/1472-6963-11-329

**Published:** 2011-11-30

**Authors:** Michèle Tony, Monika Wagner, Hanane Khoury, Donna Rindress, Tina Papastavros, Paul Oh, Mireille M Goetghebeur

**Affiliations:** 1 BioMedCom Consultants inc, Montréal, Québec Canada; 2 Workplace Safety Insurance Board of Ontario, Toronto, Ontario, Canada; 3 Toronto Rehabilitation Institute, Toronto, Ontario, Canada; 4 Centre Hospitalier Universitaire Ste Justine, Montréal Québec, Canada

## Abstract

**Background:**

Consistent healthcare decisionmaking requires systematic consideration of decision criteria and evidence available to inform them. This can be tackled by combining multicriteria decision analysis (MCDA) and Health Technology Assessment (HTA). The objective of this study was to field-test a decision support framework (EVIDEM), explore its utility to a drug advisory committee and test its reliability over time.

**Methods:**

Tramadol for chronic non-cancer pain was selected by the health plan as a case study relevant to their context. Based on extensive literature review, a by-criterion HTA report was developed to provide synthesized evidence for each criterion of the framework (14 criteria for the MCDA Core Model and 6 qualitative criteria for the Contextual Tool). During workshop sessions, committee members tested the framework in three steps by assigning: 1) weights to each criterion of the MCDA Core Model representing individual perspective; 2) scores for tramadol for each criterion of the MCDA Core Model using synthesized data; and 3) qualitative impacts of criteria of the Contextual Tool on the appraisal. Utility and reliability of the approach were explored through discussion, survey and test-retest. Agreement between test and retest data was analyzed by calculating intra-rater correlation coefficients (ICCs) for weights, scores and MCDA value estimates.

**Results:**

The framework was found useful by the drug advisory committee in supporting systematic consideration of a broad range of criteria to promote a consistent approach to appraising healthcare interventions. Directly integrated in the framework as a "by-criterion" HTA report, synthesized evidence for each criterion facilitated its consideration, although this was sometimes limited by lack of relevant data. Test-retest analysis showed fair to good consistency of weights, scores and MCDA value estimates at the individual level (ICC ranging from 0.676 to 0.698), thus lending some support for the reliability of the approach. Overall, committee members endorsed the inclusion of most framework criteria and revealed important areas of discussion, clarification and adaptation of the framework to the needs of the committee.

**Conclusions:**

By promoting systematic consideration of all decision criteria and the underlying evidence, the framework allows a consistent approach to appraising healthcare interventions. Further testing and validation are needed to advance MCDA approaches in healthcare decisionmaking.

## Background

Making decisions about the appropriate allocation of scarce healthcare resources is a necessary but difficult task. It involves consideration of a number of decision criteria, processing disparate streams of information and balancing individual and group/jurisdictional perspectives, not to mention ethical principles [1]. This complex process demands transparency, consistency, and accountability to be perceived as legitimate by the public and healthcare providers and to increase the likelihood of making good decisions [2,3].

Cost-effectiveness analysis (CEA), an economic method that aims to maximize efficiency, is the paradigm that currently dominates many healthcare policy decisionmaking processes. However, while potentially useful as a measure for productivity in healthcare, [4] sole reliance on CEA fails to address broader societal and political issues, such as disease severity, availability of alternatives, equity, and budget impact [4,5]. Even agencies that espouse the cost-effectiveness paradigm, such as NICE, acknowledge that other factors are being considered in their decisions;[5,6] however, these factors are not consistently integrated into the decisionmaking process and not revealed in a transparent fashion [5]. There are additional concerns surrounding the CEA paradigm, particularly the approach centered on the cost per quality-adjusted life-year (QALY), including methodological difficulties in valuing health states and fundamental ethical questions regarding the underlying objectives and outcomes of the approach [4,5]. Further, the complexity of some economic models can hamper understanding by the public and even by some decisionmakers of the key issues involved in the decision [6]. There is a need for a process that systematically and explicitly addresses all key factors impacting decisions, while promoting transparency and consistency in decisionmaking.

Multicriteria decision analysis (MCDA), an emerging tool in healthcare decisionmaking, goes beyond CEA by allowing systematic and explicit consideration of multiple factors that may impact the decision [1,7-10]. MCDA structures complex problems into a comprehensive set of criteria. Each criterion is weighted --a step that allows decisionmakers to clarify their fundamental objectives and perspectives [11]-- and the performance of each healthcare intervention with respect to each criterion is scored allowing identification of weaknesses and strengths [1,7-9]. Although MCDA may be perceived as not intuitive and potentially usurping decisionmaking authority, if kept simple, it facilitates an important dialog and forces decisionmakers to think hard about and clearly express what they value, why they value it, and in what context they value it.

In addition to the emergence of MCDA, recent decades have seen growth in the field of Health Technology Assessment (HTA) [12]. With a mission "to assist and advise healthcare decisionmakers in defining health policies at all levels", [13] HTA acts as a bridge between evidence and decisionmaking to ensure better synthesis, communication and dissemination of information [14]. It is now widely recognized that to fulfill its mission, in addition to clinical and economic factors, HTA needs to address social, organizational, ethical and legal dimensions of health technology [15-18]. Current efforts to develop international standards for HTA reports [19,20] point to the need for a structured format that can provide full access to the underlying evidence, thereby enhancing transparency and usability of the report to decisionmakers and stakeholders.

MCDA, HTA, and knowledge translation have a common objective: enlightened and evidence-based healthcare decisionmaking. Reflection on the drivers behind healthcare decisions is essential to ensure that these fields of research are aligned with the practical needs of decisionmakers. A pragmatic framework (EVIDEM), proposes a standard set of criteria with detailed methodology i) to provide validated synthesized evidence for each criterion (by-criterion HTA report), and ii) to systematically consider each criterion using a MCDA Core Model and a Contextual Tool [21]. Criteria were defined based on an extensive analysis of the literature and decision processes around the world, as well as discussion with stakeholders; tools were developed to stimulate reflection on priorities, to support systematic deliberation and to facilitate pragmatic knowledge transfer [21,22].

The EVIDEM framework underwent proof-of-concept evaluation by a panel composed of a broad range of Canadian stakeholders appraising 10 medications [23]. As a support for policy decisionmaking, the framework was field-tested with the reimbursement advisory committee of a private health plan in South Africa using a cervical cancer screening tool as a case study [24]. For clinical decisionmaking, the framework was tested by a panel of pediatric endocrinologists and other Canadian stakeholders using growth hormone for Turner syndrome as a complex case study with far reaching ethical issues; this study led to further development of the framework and explicit integration of ethical and system-related criteria into the decision process [22]. The objective of this study was to field-test a MCDA-based framework (EVIDEM), explore its utility to a drug advisory committee and test the stability of estimates over time using tramadol for chronic non-cancer pain (CNCP) as a case study relevant to their context.

## Methods

### Study design

**Figure 1:**
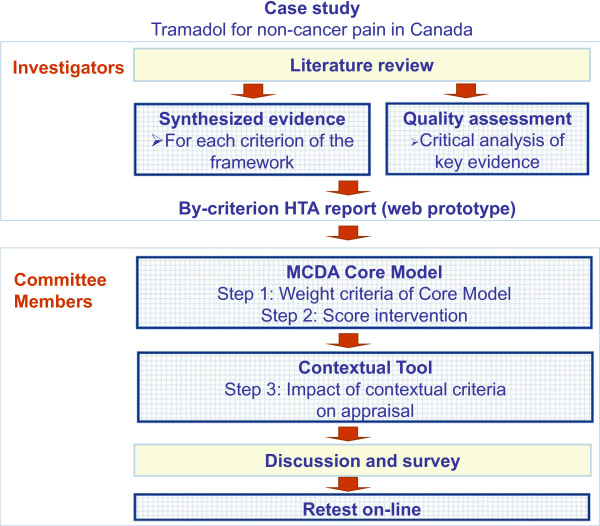
**Study plan**.

In a move to build on existing decisionmaking processes, the EVIDEM framework was field-tested by the Drug Advisory Committee of the Ontario Workplace Safety Insurance Board (WSIB), which provides benefits, including healthcare benefits, to workers suffering injury or illness directly related to work. Tramadol for CNCP was selected by the committee as a relevant case study to the context of the population covered by the WSIB. The study design is presented in Figure 1. Based on an extensive literature review, a structured HTA report on tramadol for CNCP was produced, tailored to provide detailed information, as available, for each criterion of the EVIDEM framework, i.e., 14 criteria for the MCDA Core Model and 6 qualitative criteria for the Contextual Tool [21,22]. Synthesized data integrated in the MCDA Core Model and the Contextual Tool is referred to as the "by-criterion HTA report". During workshop sessions, committee members tested the framework in three steps by assigning: 1) weights to each criterion of the MCDA Core Model representing individual perspective; 2) scores for tramadol for each criterion of the MCDA Core Model using synthesized data (by-criterion HTA report); and 3) qualitative impacts of the Contextual Tool criteria on the appraisal. Utility and reliability of the approach were explored through discussion, survey and test-retest.

### By-criterion Health Technology Assessment report

An extensive analysis of the published and grey literature was performed to identify relevant data for each criterion of the framework. Databases and sources searches, including PubMed, EMBASE, Cochrane, Disease Association web sites (Canadian Pain society; Chronic Pain Association of Canada), and websites of the Agency for Healthcare Research and Quality (AHRQ), the National Institute for Clinical Excellence (NICE), the Canadian Agency for Drugs and Technologies in Health (CADTH), and the World Health Organization (WHO), were completed by hand searching of bibliographies. Search terms included: tramadol, opioid/NSAID/COX-2, chronic pain, chronic non-cancer pain, osteoarthritis/low back pain/neuropathic pain/fibromyalgia, randomized controlled trial/non- randomized trial, WOMAC/Pain and sleep Questionnaire/Chronic Pain Sleep Inventory, abuse/dependence, quality of life/QoL/HRQoL, epidemiol*/prevalence/incidence, mortality, guideline/recommendation/clinical practice, pain management, patient- reported outcomes*/PRO, burden, depression/anxiety, cost*/econom*, productivity, ethic*.

To provide the most relevant data to the committee, evidence in the Canadian context was researched, supplemented with information from other countries. Disease information was obtained from prominent reviews and epidemiological studies in Canada and elsewhere. Analyses of the limitations of current options and of current clinical guidelines for pain management in Canada and elsewhere were performed. Clinical, safety and patient-reported outcomes (PRO) evidence for tramadol and comparators for the most important primary outcomes [25,26]) were obtained from active-controlled randomized clinical trials (RCTs) and product monograph. Although inclusion of observational studies can supplement evidence from RCTs, [27,28] no such studies were identified. Economic data was obtained from the literature supplemented by analyses provided by the health plan. Critical analysis of key clinical and economic studies was performed using the EVIDEM tools described previously [21] to explore the quality of evidence. For the contextual criteria, scientific and grey literature was searched for information on ethical, historical, and contextual aspects of tramadol and opioid treatment.

Data collected was synthesized for each criterion of the framework following the EVIDEM methodology [21] based on HTA best practices recommendations (Busse et al., 2002 [29]). To streamline access to evidence and limit data overload, an interactive web prototype (Tikiwiki v3.0) was developed to provide highly synthesized data for each criterion ('Lite' version, for a quick grasp of issues), hyperlinked to a version with more details ('Full' version) with further hyperlinks to the full text sources from which data was extracted. The web prototype was also designed to allow committee members to enter weights, scores and impacts for each criterion for online appraisal of the selected medicine.

### Field-testing with committee

To explore individual perspectives, during the workshop session (test), each member of the committee (n = 9) were instructed to assign weights individually (on a scale of 1-low to 5-high) to each criterion of the MCDA Core Model, from their perspective in the context of the health plan. For consistency across interventions, committee members were instructed to attribute these weights independently of the intervention; these weights are expected to be defined once and then used throughout appraisals.

Time was allotted on an as-needed basis. This was followed by a period of discussion on each criterion, and committee members were allowed to modify their weights, on an individual basis.

To appraise the intervention, committee members were instructed to score individually (on a scale of 0 to 3) each criterion of the MCDA Core Model, using evidence synthesized for each of them (by-criterion HTA report). This was followed by a period of discussion on each criterion, and committee members were allowed to modify their scores, on an individual basis.

Committee members then explored the six contextual criteria and assigned the type of impact (negative, none or positive) each criterion would have on the appraisal of tramadol, using the colloquial and scientific evidence integrated into the Contextual Tool.

Feedback on the framework, included criteria and process was collected during discussion periods at the first workshop and at a follow-up workshop, and from a questionnaire administered during the follow-up workshop. To explore reliability, a retest was performed at least two weeks after the last session either using the web-based prototype on-line or a hardcopy document.

### Data collection and analyses

For the test, weights, scores and impacts were obtained using the hardcopy documents distributed to committee members and entered into Microsoft Excel software. Data entered on-line by panelists (retest) was recorded in a MySQL database and transferred to the Excel software, which was then used to perform statistical analyses.

Descriptive statistics were used and mean ± standard deviations (SD) were reported for weights and scores.

The MCDA value estimate of the perceived value of tramadol for the treatment of CNCP was obtained by applying an MCDA linear additive model to combine weights and scores [30]. The MCDA value estimate is anchored on a scale of no value (0, e.g., minor symptom relief for a rare, mild condition with numerous alternative treatment options that provides worse efficacy, safety and quality of life and produces no public health benefits but results in major additional spending) to maximum value (1, e.g., cure for an endemic severe disease with limited treatment alternatives that provides significant improvement in efficacy, safety and quality of life, and produces major public health benefits and healthcare savings) [21].

$$\text{V}=\overset{\text{n}}{\underset{x=1}{\sum }}{\text{V}}_{x}=\overset{n}{\underset{x=1}{\sum }}\left(\frac{{\text{W}}_{x}}{\overset{n}{\underset{x=1}{\sum }}{\text{W}}_{x}}\;\times {\text{S}}_{x}\right)$$

The estimate was thus calculated as the sum of value contribution (Vx) of combined normalized weights (Wx) and scores (Sx) for applicable criteria (n = 14) of the MCDA Core Model. Calculations were performed for each committee member (e.i., combining weights and scores for each individual) and then averaging the MCDA value estimates for the 9 committee members.

Agreement between test and retest data was analyzed by calculating intra-rater correlation coefficients (ICCs) for weights, scores and MCDA value estimates. One type of ICCs was calculated following Shrout and Fleiss (1979) [31] methods and classification: the ICC (3, 1) which is based on a two-way mixed analysis of variance (ANOVA) model (general effects of the test and the retest were assumed to be fixed). In addition, the proportion of data pairs that did not differ between test and retest, that differed by 1 point, and that differed by 2 points, was calculated for weights and scores.

Inter-rater correlation coefficients were not calculated since the tool is designed to capture individual perspectives, which are expected to vary among individuals.

## Results

### By-criterion Health Technology Assessment report

Synthesized data was based on 69 references covering all the criteria of the framework. The highly synthesized version of the by-criterion HTA report with assessment scales is reported in Additional file 1. The web HTA report with all levels of synthesis is available online [32].

The report provides an overview of tramadol, a weak opioid indicated for the treatment of CNCP, and the context of this treatment in Canada. In summary, CNCP is a disabling condition affecting 25% of the Canadian population [33]. Efficacy and safety of current treatments are limited due to ceiling effects and the potential for organ damage [34-46].. In randomized trials, tramadol significantly reduced pain intensity from baseline, but this reduction was not significantly different from NSAIDs, COX-2 inhibitors and opioids [47-51]. The tolerability of tramadol is comparable to that of other analgesics, [47,49,51-56] but drug abuse may be lower with tramadol than with other opioids [57,58]. Significant changes from baseline were observed for patient-reported outcomes, which were not significantly different than those reported for comparators, including placebo [47,48]. Relevance and validity of trial data was limited by: the trial population (mostly older patients with osteoarthritis (OA) not relevant to WSIB); trial durations (a few weeks), high attrition rates in 3 trials and unclear reporting in 2 trials.

Economic data indicated that, based on a daily cost of tramadol of $2.24-2.27 (comparators costs range $0.11 to $6.61), reimbursement of tramadol would result, depending on models assumptions, in savings of 0.32% of additional expenditures and 0.27% of the current budget for pain analgesics for the health plan budget [unpublished data]. A Dutch cost-minimization analysis indicated that, compared to NSAIDs, tramadol would result in savings of $534 to $574 per patient over six months when considering adverse events associated with NSAIDs [59]. Relevance and validity of this study were limited by the setting and OA population, a short time horizon and costs considered (e.g., no costs from productivity losses). To stimulate reflection and discussion in the context of the health plan, information was provided in the Contextual Tool on: the utility of the treatment, [60,61] its efficiency and potential opportunity costs, fairness and access to care for opioid analgesics, [61,62] risk of abuse, [57,63,64] pressures from the Canadian Pain Society to keep tramadol out of the controlled drug schedule, [65,66] historical reviews of the WHO on tramadol [67] and recommendations on tramadol from Canadian agencies. This report was used for field-testing of the framework with the committee.

### Field-testing with committee

#### Decision criteria and committee perspective - weights

**Figure 2:**
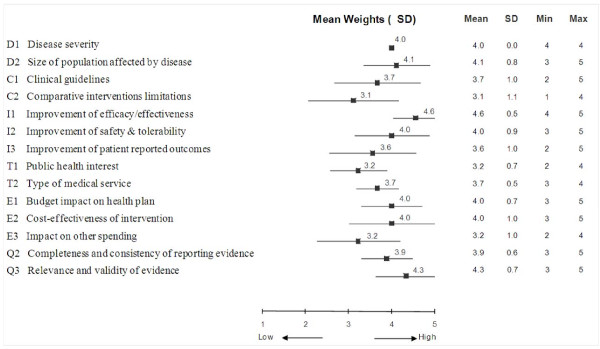
**Mean weights for decision criteria of the MCDA Core Model from the drug advisory committee**. A five-point weighting scale was used with 1 for lowest weight and 5 for highest weight.

Independently of the specific case of tramadol, each member of the committee assigned weights on a scale of 1 to 5 to the criteria of the MCDA Core Model to express individual perspectives. Mean weights and standard deviations are reported in Figure 2. At the committee level, the greatest importance was given to the criteria "Improvement of efficacy/effectiveness" (4.6 ± 0.5) and "Relevance and validity of evidence" (4.3 ± 0.7). The least important criterion was "Comparative interventions limitations" (3.1 ± 1.1). A perfect consensus among committee members was observed for importance of the criterion "Disease severity" (SD: 0). Weights for "Improvement of efficacy/effectiveness" and "Type of medical service" also varied little among committee members (SD: 0.5). The largest divergence of opinions was recorded for the criterion "Comparative intervention limitations" (SD: 1.1).

Survey data and discussion revealed that committee members felt that most of the criteria considered in the MCDA Core Model and the six contextual criteria were relevant in their context and should be systematically considered. Although the criterion "Disease severity" was considered important by the committee (weight: 4.0 ± 0), the necessity of this criterion was discussed; some committee members noted that because of the type of conditions covered by the health plan (i.e., work-related illness or injury), scores for criterion "Disease severity" may always be high in the committee context. However, it was also noted that some of the population covered by the health plan suffer from really severe diseases and that the framework would capture this aspect. "Public health interest" was considered by some committee members as irrelevant in their context, and there was some controversy on considering the criterion "Political/historical context".

#### MCDA Core Model - scores for tramadol

**Figure 3:**
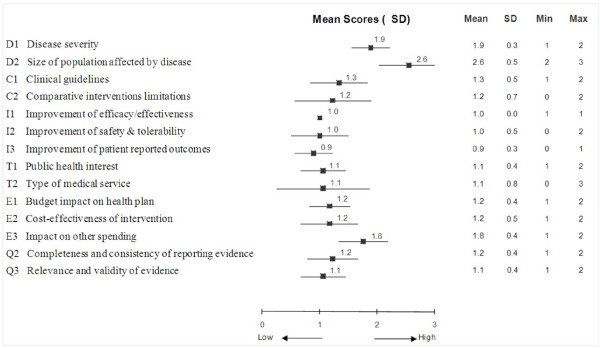
**Mean scores for decision criteria of the MCDA Core Model for the appraisal of tramadol by the drug advisory committee**. A four-point scoring scale was used with 0 for lowest score and 3 for highest score.

Using synthesized data integrated in the MCDA Core Model and the Contextual Tool (by-criterion HTA report) (Additional file 1), committee members assigned scores to appraise tramadol for each criterion (Figure 3). The highest scores were assigned to the criteria "Size of population affected by disease" (2.6 ± 0.5, on a scale of 0 to 3), "Disease severity" (1.9 ± 0.3) and "Impact on other spending" (1.8 ± 0.4). In contrast, low scores were given to "Improvement of patient-reported outcomes" (0.9 ± 0.3), "Improvement of efficacy/effectiveness" (1.0 ± 0.0) and "Improvement of safety & tolerability" (1.0 ± 0.5). The committee demonstrated a unanimous agreement concerning the performance of tramadol with respect to "Improvement of efficacy/effectiveness" (SD: 0.0). In contrast, differences of 2 and 3 points (on a scale of 0 to 3) were observed for "Type of medical service" (SD: 0.8) and "Comparative intervention limitations" (SD: 0.7), respectively. Committee members indicated that average scores for the MCDA criteria presented in Figure 3 had good face validity and represented their opinion of tramadol.

The scoring direction for the criterion "Size of the population", which stipulates a higher score for interventions for conditions with high prevalence/incidence, was questioned as committee members felt that interventions for rare conditions should not be valued lower than those for more common conditions. Committee members also raised the importance of patient preferences and their perspective on the disease, which could be more explicitly considered under the criterion "Patient-reported outcomes". When evaluating the criterion "Improvement of efficacy/effectiveness", committee members noted that while they consider RCTs are the primary sources of data on efficacy, non-randomized studies can sometimes be useful for providing supplementary information on safety and real-life effectiveness.

#### MCDA Core Model - value estimate for tramadol

**Figure 4:**
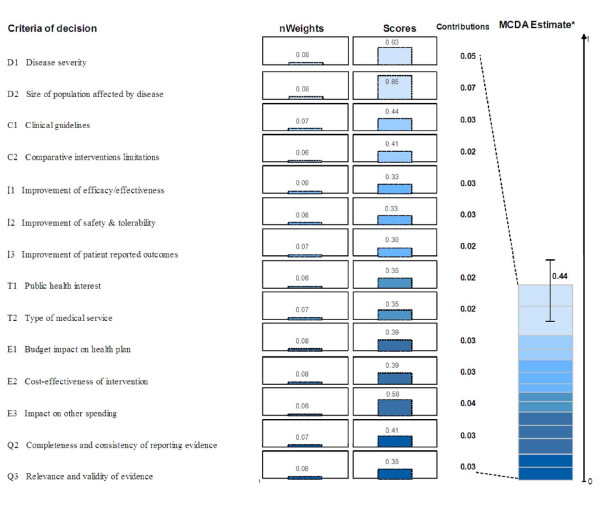
**MCDA value estimate of tramadol for chronic non-cancer pain**. Weights were normalized across the 14 criteria and scores are presented on a scale of 0 to 1. *MCDA value estimate was obtained using a linear model combining normalized weights and scores for each decision criterion. For an intervention to achieve close to 1 on this scale, it would have to cure an endemic disease, demonstrate major improvement in safety, efficacy, and patient-reported outcomes compared to limited existing approaches and result in major healthcare savings.

The MCDA value estimate for tramadol, which integrates perspectives of committee members (weights) and performance measurements (scores), was calculated for each member of the committee by combining normalized weights and scores using a linear additive model. At the individual level, the MCDA value estimates ranged between 0.36 and 0.61 on a scale of 0 to 1. For the group, the overall MCDA value estimate was 0.44 ± 0.07. More than one quarter (26%) of the estimated value of tramadol was derived from the two criteria of the "Disease impact" cluster ("Disease severity" and "Size of population") (Figure 4). Relative contributions of the other criteria to the MCDA value estimate for tramadol ranged between 5% and 8%.

Committee members indicated that quantification of evidence and interpretation of the MCDA value estimate of 0.44 was challenging and required detailed explanations on mathematical calculations used in the framework. It was deemed difficult to interpret the end result of the MCDA Core Model. Scaling with other drugs appraised by the committee (preferably with widely differing MCDA value estimates) would be needed to clarify the meaning of MCDA value estimates and get a better grasp of how the methodology can be used for ranking interventions. Some committee members expressed reluctance to base decisions on cut-off values, which is not the objective of the framework, as it allows for qualitative contextual considerations to modulate the estimate (see section below - Contextual Tool). In addition, the framework is meant to support the evaluation and deliberation leading to the decision, which was deemed very important by the committee.

#### Contextual Tool - impacts of contextual criteria on tramadol appraisal

**Figure 5:**
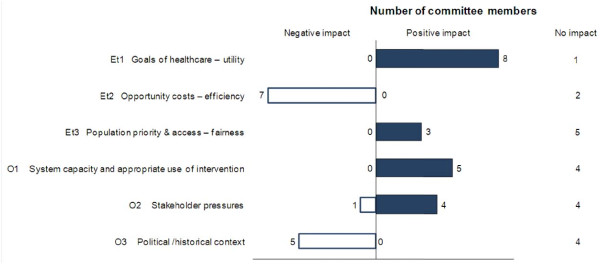
**Impact of contextual criteria on tramadol appraisal by the drug advisory committee**.

Based on the synthesized information collected for the six contextual criteria (Additional file 1) and their own understanding of the context, committee members considered what type of impact (positive, negative or neutral) each contextual criterion would have on the appraisal of tramadol. The distribution of these impacts within the committee is shown in Figure 5.

For most committee members (8 out of 9), "Goal of healthcare - utility" had a positive impact on the appraisal of tramadol, since relieving pain is aligned with the goals of healthcare in general and the WSIB in particular. Regarding the criterion "System capacity and appropriate use of intervention" opinions were divided (5 indicated a positive, 4 a neutral impact), as committee members indicated: that "abuse with tramadol exists"; that there is a "need for abuse data for the true comparator (e.g., codeine) rather than with more potent hydrocodone which is a misleading comparison"; but that there is a "growing concern/burden about opiate abuse and any drug with potential to decrease that burden deserves special consideration".

Consideration of "Opportunity cost - efficiency" had a negative impact on the appraisal for most (7 out of 9) committee members as it was noted that "tramadol is more expensive than comparable medicines". For more than half of committee members (5 out of 9), "Political/historical context" had a negative impact on the appraisal of tramadol. As stated by one committee member "negative recommendations from other agencies have a negative impact on tramadol".

Regarding "Population priority and access and fairness", 5 out of 8 committee members indicated that this criterion had no (neutral) impact on the appraisal. One member did not provide data for this criterion and indicated that it was not clear how to consider it, highlighting the need for thorough reflection on this criterion which is meant to elicit discussion on priorities of the health plan. Impacts were mixed for "Stakeholders pressures".

Explicit consideration of the six contextual criteria forced reflection on a broad range of issues, which were considered to have mixed impacts on the overall appraisal of tramadol.

### Feedback from committee on overall approach

Participants indicated that the framework is a good point of departure to help lead group discussions and to ensure systematic, transparent and consistent consideration of important elements that may affect decisionmaking. To cite one committee member: "EVIDEM is a good tool in the sense that it forces each member to think and weight aspects that otherwise would not have been considered". Another committee member felt that it may help validate decisions and demonstrate transparency to stakeholders. Some members voiced concern about the amount of work involved in developing the by-criterion HTA report. Others questioned the complexity of the tool and terminology used. One committee member stated that the framework adds clarity to decisionmaking but repeated use may be required to fully capture its utility and get acquainted with its application.

### Exploration of reliability

Weighting and scoring during the test and the retest led to the same mean MCDA value estimate of 0.44 (Table 1). The ICC (3, 1) for these data was 0.698, indicating fair to good reproducibility. ICC (3, 1) values of 0.683 for weights and of 0.676 for scores were also a sign of good reliability of test-retest weights and scores.

**Table 1:** Agreement at the individual level between test-retest for weights, scores and MCDA value estimates for tramadol

	Weights	Scores	Value Estimates
Number of test-retest pairs	126*	126*	9
Mean of test data	3.81	1.31	0.44
Mean of retest data	3.81	1.33	0.44
ICC (3, 1)	0.683	0.676	0.698
Proportion of pairs with no test-retest difference (%)	65.1	78.6	NA
Proportion of pairs with test-retest difference of 1 point (%)	31.7	19.8	NA
Proportion of pairs with test-retest difference of 2 points (%)	3.2	1.6	NA

*9 evaluators × 14 MCDA criteria

NA: Not applicable

ICC: intra-class correlation coefficient, defined according to Shrout and Fleiss (1979) [31]

Between test and retest, 65.1% of weights were identical, 31.7% differed by 1 point (on a scale of 1 to 5) and 3.2% differed by 2 points. The greatest inconsistency between test and retest weights was recorded for the cluster "Context of intervention" (38.9% identical) and the least for the clusters "Disease impact" and "Intervention outcomes" (77.8% identical).

Scores exhibited better consistency between test and retest than weights: 78.8% were identical, 19.8% differed by 1 point (on a scale of 0 to 3) and 1.6% differed by 2 points. The greatest difference between test and retest was recorded for clusters "Context of intervention" and "Type of benefits" (61.1% identical) and the least for clusters "Disease impact" and "Quality of evidence" (88.9% identical).

## Discussion

The framework was found useful by the drug advisory committee in supporting systematic consideration of a broad range of criteria to promote a consistent approach to appraising healthcare interventions. Directly integrated in the framework as a "by-criterion " HTA report, synthesized evidence for each criterion facilitated its consideration, although this was sometimes limited by lack of relevant data. This is in agreement with previous studies in which committee members and panelists indicated that the framework was a useful approach to systematic consideration of all aspects of decision, facilitating consistency, transparency and clarity of appraisal and decisionmaking [22,24]. Test-retest analysis showed fair to good consistency of weights, scores and MCDA value estimates at the individual level (ICC ranging from 0.676 to 0.698), thus lending some support for the reliability of the approach. Overall, committee members endorsed the inclusion of most framework criteria and revealed important areas of discussion, clarification and adaptation of the framework to the needs of the committee.

The EVIDEM framework is aligned with the four key features of HTA identified by Battista et al.:[14] policy orientation; interdisciplinary content and process; synthesis of information; and facilitation of dissemination and communication of information. The framework proposes a comprehensive set of criteria to promote systematic and explicit consideration of all aspects of decision, including ethical and system-related issues, considered vital for HTA to fulfill its mandate [68]. Testing revealed the need for adjusting criteria and processes to ensure MCDA approaches can support existing processes and meet the needs of decisionmakers. The framework is meant to be adapted and some tools have been developed since to facilitate adaptation of the framework based on collaborative development with users and researchers [69].

Standardized procedures for gathering, synthesizing, distilling, and presenting information to inform each criterion, an integral part of the framework, can also contribute to enhancing consistency of the decisionmaking process. As advocated by Straus, [70] Robeson [71] and their coworkers, data was made available on the web in two hyperlinked levels: a 'lite' version with data distilled into key messages --to facilitate knowledge transfer and to reduce the time required to integrate the information presented-- and a comprehensive, detailed synthesis with full access to underlying sources of evidence. Although presenting the data at these two levels of synthesis was found to be helpful by some committee members, others would have preferred the 'lite' version to provide more details. Thus, while the comprehensive version of the by-criterion HTA report should always serve as a basis, subsequent knowledge distillation may need to be further optimized to strike the right balance between conciseness and detail. Collaborative development is ongoing to further advance the methodology to synthesize data for each criterion and for different levels of details.

The weighting exercise and following discussion revealed the different perspectives of committee members as captured by the large SDs for some criteria. Variations may also be due to different understanding of the criteria, although detailed definitions were provided and were further clarified during discussions. For consistency across interventions, it is recommended that these weights be attributed once and then used throughout appraisals. Although at the individual level, relative weights vary largely between criteria, once weights are averaged at the committee level, extremes disappear and weights tend to be less distinguishable. Exploration of how the weight elicitation methods (e.g., analytical hierarchy process [AHP], Simple Multi-Attribute Rating Technique [SMART], point allocation, ranking)[72], impact weight attribution and the overall MCDA value estimate is ongoing to further advance the approach and provide additional tools to adapt the framework to the preferences and needs of users.

Interpretation and utility of the MCDA value estimate (i.e., the figure 0.44) was found challenging by committee members. Indeed, the MCDA value estimates are meant to be used in a comparative manner for ranking healthcare interventions, which was beyond the scope of this case study. An MCDA model adapted from the EVIDEM framework by a district health board uses such an approach to systematically evaluate and prioritize a wide range of healthcare interventions (Sharon Kletchko, MD, personal communication 2011). Although basic principles are explicit, interpretation of the MCDA scale requires acquaintance with the broad range of criteria that are incorporated in a single number. For example, an intervention with a score close to 1, which would constitute a high-end reference point, would have to be something like: a cure for an endemic severe disease with limited or no alternatives that provides significant improvement in efficacy, safety and quality of life, and produces major public health benefits and healthcare savings [21]. A pilot study testing the framework with 13 Canadian healthcare stakeholders showed that the MCDA value scale had discriminating properties, with mean estimates ranging from 0.42 to 0.64 across 10 medicines [23]. However, it should be kept in mind that MCDA value estimates are not meant to be used in a prescriptive fashion, but rather as "a framework conducive for focused discussion."[73] MCDA value estimates can serve as a basis for establishing a ranking scheme, [9] which can be modulated by ethical and context related considerations. This is often done implicitly in healthcare decisionmaking and is meant to be facilitated by the Contextual Tool. It should also be noted that MCDA value estimates obtained by applying this framework are committee-specific since they reflect the individual perspectives of committee members as captured by weights.

Although there are numerous approaches to improve healthcare coverage decisionmaking --including the prevailing cost-effectiveness paradigm, program-budgeting and marginal analysis, and the HTA Core Model [74-76], for example -- there is no accepted and validated way to identify successful evaluation and decisionmaking and still less consensus concerning the best framework to support decisionmaking [77] or even the most reliable process for weight elicitation [78]. In that sense, our study suffered limitations common to other studies on the validity of decisionmaking approaches. Exploration of reliability revealed good consistency at the individual level which constitutes a first positive step. Higher consistency with scores, which are based on evidence, than with weights, which are based on individual perspective, were observed in this study and may reveal the difficulty to explicit its one's perspective. This might stem from the fact that perspectives are often implicit and that decisionmakers need to reflect on the implications of the criteria. As for every evaluation of healthcare interventions, the information presented on the case study was limited by the information available at the time of the study. Nevertheless, this field study has shown that, through bridging HTA and MCDA, the EVIDEM framework can support appraisal in practice, particularly by promoting systematic consideration of a comprehensive set of carefully selected criteria. A strength of the framework also lies in the acknowledgment and incorporation into its application that decisionmaking is a "fundamentally value-laden enterprise" [68]. These features are combined with firm grounding in scientific evidence, which includes rigorous synthesis and quality assessment, to make the committee's deliberative process as well-informed, comprehensive and explicit as possible. The framework promotes a consistent approach to decisionmaking that can help legitimize decisions and be aligned with the A4R framework set forth by Daniels [79,80].

## Conclusions

In a field test with a public health plan drug advisory committee, the EVIDEM framework supported appraisal and deliberations in practice by bridging HTA and MCDA. Feedback from some committee members confirmed that the framework promoted the explicit consideration of a wide range of criteria relevant to decisionmakers. Further field testing is required to establish a frame of reference for appraisal outcomes, optimize and adjust its use in practice, and establish consistency. Further research is needed to collaboratively advance pragmatic MCDA-based frameworks for appraisal of healthcare interventions, decisionmaking, priority setting and pragmatic knowledge translation.

## List of abbreviations used

AHRQ: US Agency for Healthcare Research and Quality; ANOVA: Analysis of variance; CEA: Cost-effectiveness analysis; CNCP: Chronic non-cancer pain; EVIDEM: Evidence and Value: Impact on DEcisionMaking; H2RA: Histamine receptor antagonist; HRQOL: Health-related quality of life; ICC: Intra-rater correlation coefficients; MCDA: MultiCriteria Decision Analysis; NSAID: Nonsteroidal anti-inflammatory drug; OA: Osteoarthritis; PRO: Patient-reported outcomes; QALY: Quality-adjusted life-year; QoL: Quality of life; RCT: Randomized clinical trials; WOMAC: The Western Ontario and McMaster Universities Arthritis Index; WSIB: Ontario Workplace Safety Insurance Board.

## Competing interests

The authors declare that they have no competing interests.

## Authors' contributions

MMG, DR & MW designed the study and reviewed the HTA report and MCDA analyses. MT performed data collection and analyses. HK & MW participated in data analyses. TP and PO participated in data collection and reviewing tools and synthesized evidence. MT, MMG, MW and HK drafted the manuscript. All authors reviewed the manuscript and approved the final version.

## Pre-publication history

The pre-publication history for this paper can be accessed here:

http://www.biomedcentral.com/1472-6963/11/329/prepub

## Supplementary Material

Additional file 1 **By-criterion health technology assessment report on tramadol ('Lite' highly synthesized version)**. References for each statement and links to sources are available on the web version of this report [32]. Click here for file
