# Supplementary material for: Bridging health technology assessment (HTA) with multicriteria decision analyses (MCDA): field testing of the EVIDEM framework for coverage decisions by a public payer in Canada
Source: BMC Health Serv Res. 2011 Nov 30;11:329. doi: 10.1186/1472-6963-11-329 (PMC3248909; doi:10.1186/1472-6963-11-329)
Supplement: Additional file 1 — By-criterion health technology assessment report on tramadol ('Lite' highly synthesized version). References for each statement and links to sources are available on the web version of this report [32]. [file 1472-6963-11-329-S1.DOC]

## Additional file 1 - By-criterion health technology assessment report on tramadol (‘Lite’ highly synthesized version)

References for each statement and links to sources are available on the web version of this report.[32]

| **Overview** | | |  |
| --- | --- | --- | --- |
| - **Intervention:** Tramadol - **Disease:** Chronic non-cancer pain (CNCP) - **Setting:** Canada (WSIB) | | - **Drug class**: non-conventional weak opioid with dual mechanism of action (weak mu-opioid effect of M1 metabolite, and weak monoaminergic effect) - **Indication**: moderate to moderately severe pain - **Administration**: tablets, once daily (slow-released tramadol – Ralivia, Tridural, Zytram); max 8 tablets daily (short acting Tramacet) - **Intervention duration**: several days or more; head to head trials: 4 to 12 weeks - **Comparator(s)**: placebo, NSAIDs, COX-2 inhibitors, opioids - **Economic burden of illness:** osteoarthritis - annual cost: $3.59 billion; chronic low back pain - annual cost per patient (Netherlands): $13,770; fibromyalgia - annual cost per patient: $16,134 |  |
| **MCDA Core Model criteria** | | **Highly synthesized information** | **Scoring intervention** |
| **Disease impact** | | |  |
| D1 | Disease severity | Chronic non-cancer pain includes nociceptive (tissue damage) and neuropathic (nerve pathology) pain; 37% of low back pain of neuropathic origin.  Disabling condition interfering with activities of daily living (28% patients), work (lost income in 49% patients) and education.  Associated with depression and/or anxiety (40% patients) | Not severe  0 1 2 3  Very severe  ***Comments*** |
| D2 | Size of population | **Prevalence/Incidence**: Canadian pain study 2004 (N=1055 in general population): 25% with chronic pain (88% moderate or severe); mean duration 9.8 years | Very rare disease  0 1 2 3  Common disease  ***Comments*** |
| **Context of intervention** | | |  |
| C1 | Clinical guidelines | **Canadian Pain Society guidelines**:   - ***Chronic non-cancer pain (2002)***: no mention of tramadol - mild to moderate: 1st line non-opioid, 2nd line opioid (moderate to severe pain: switch to opioid earlier) - ***Chronic neuropathic pain(2007)***: 3^rd^ line tramadol/conventional opioid   **Other countries recommending tramadol for:**   - ***Osteoarthritis***: 2nd line, USA - ***Chronic low back pain***: 2nd line, USA and Europe - ***Fibromyalgia***: 2nd line, USA | Not recommended  0 1 2 3  Strong recommendation  ***Comments*** |
| C2 | Comparative interventions limitations | **NSAIDs and COX-2 inhibitors**: ceiling effect in pain reduction; organ damage after long term use (cardiac and renal; and gastric for NSAIDs)  **Opioids**: not efficacious in pain of neuropathic origin; gastrointestinal (constipation, nausea, vomiting), respiratory (respiratory depression) and neurologic (dizziness, somnolence) side effects; risk of tolerance/dependence/abuse  **Specific to neuropathic pain:**   - Anticonvulsants (e.g.,gabapentin, pregabalin): sedation, dizziness, ataxia, somnolence, confusion - Tricyclic antidepressants (e.g., amytriptiline): sedation, dry mouth, constipation, orthostatic hypotension, weight gain - SNRIs (e.g.,duloxetine, venlafaxine): nausea, dyspepsia, sweating, somnolence and insomnia - SSRIs (e.g. fluoxetine): agitation, anxiety, sleep disturbance, tremor, sexual dysfunction and headache | No or very minor limitations  0 1 2 3  Major limitations  ***Comments*** |
| **Intervention outcomes** | | |  |
| I1 | Improvement of efficacy/ effectiveness | *Trial results obtained with WOMAC and other scales were recalculated to be expressed on a normalized scale from 0 (minimum improvement) to 100 (maximum improvement). If not mentioned, no significant difference*  **5 Head to head randomized controlled trials** (osteoarthritis, low back pain & other; 4 to 12 weeks; N =108 to 1001; 1 in Canada, 4 in USA):   - ***Pain intensity reduction from baseline***: tramadol: 15-24; diclofenac: 16; celecoxib: 26 (*P*=0.05 vs placebo); placebo: 19 *(significant difference for all vs baseline)* - ***Pain intensity reduction after 6 hrs***: tramadol: 21; codeine: 18   **Placebo randomized controlled trials (Cochrane reviews)**:   - ***Pain intensity reduction versus placebo***: - 8.5 (osteoarthritis, 3 trials, N= 92 to 307) - 10.8 (chronic low back pain, 3 trials, N= 254 to 336) - ***Patients achieving 50% pain relief:*** 63% tramadol vs 37% placebo (neuropathic pain, 3 trials, N=67 to 127) | Lower than comparators  0 1 2 3  Major improvement  ***Comments*** |
| I2 | Improvement of safety & tolerability | **Summary of common AEs** (> 5% of patients in RCTs and frequency > twice of placebo in tramadol product monographs**):**   - nausea: tramadol 10–24%; codeine 5–19%; diclofenac 11% - somnolence: tramadol 7–18%; codeine 3–24%; diclofenac 8% - dizziness: tramadol 7–24%; codeine 5–14%; diclofenac 18% - constipation: tramadol 7–21%; codeine 10–21%; diclofenac 15% - vomiting: tramadol 4–15%; codeine 1–7%; diclofenac 5% - dry mouth**:** tramadol 5%, diclofenac 7% - sweating: tramadol 1–15%; codeine 1%; diclofenac 0%   **Warnings tramadol:** Seizure risk; anaphylactoid reactions, drug abuse, addiction and dependence; withdrawal symptoms; risk of overdosage; increased intracranial pressure or head trauma; respiratory depression  **Drug abuse**: ***12 month study***: tramadol 2.7%, NSAIDs: 2.5%; hydrocodone: 4.9%  ***Surveillance study***: tramadol: 0.5–2 cases/100,000 patients (over 10 years); oxycodone: at least 5/100, 000 cases (over 3 years) | Lower than comparators  0 1 2 3  Major improvement  ***Comments*** |
| I3 | Improvement of patient reported outcomes | *Trial results obtained with WOMAC and other scales were recalculated to be expressed on a normalized scale from 0 (minimum improvement) to 100 (maximum improvement). If not mentioned, no significant difference*  **3 Head to head RCTs** (osteoarthritis studies; 6 to 12 weeks, N=108 to 1001, 1 in Canada, 2 in USA):   - ***Physical function improvement from baseline:*** tramadol: 15-21, diclofenac: 15, celecoxib: 25 (*P*=0.05 vs placebo), placebo: 17 - ***Stiffness reduction from baseline:*** tramadol: 17, diclofenac: 18 - ***Quality of sleep improvement from baseline:*** tramadol: 10, diclofenac: 8   *(P< 0.05 for all vs baseline; in 1 trial, no numbers reported for stiffness and quality of sleep but significance reported for tramadol300 mg vs placebo and for celecoxib vs placebo)* | Lower than comparators  0 1 2 3  Major improvement  ***Comments*** |
| **Type of benefit** | | |  |
| T1 | Public health interest | **Risk of depression**: The degree of depression improvement correlates with the amount of pain relief. Pain relief with tramadol may therefore have an impact on the risk of depression (no data available) | No risk reduction  0 1 2 3  Major risk reduction  ***Comments*** |
| T2 | Type of medical service | **Tramadol** produces symptom relief (20 points from baseline on a scale from 0 to 100) and may improve physical function, but does not cure pain | Minor service  0 1 2 3  Major service (*e.g.cure)*  ***Comments*** |
| **Economics** | | |  |
| E1 | Budget impact on health plan | **Average daily cost per patient (based on historical claims data):**  **Tramadol**: $2.24–2.27 (long-acting) to $2.91 (short acting)  **Comparators**:   - NSAIDs: $0.11–0.80 - COX-2 inhibitor: $1.58 - Opioids: $0.67–6.61 - Antidepressants: $0.61–8.77* - SNRIs: $2.66–8.77* - Anticonvulsants: $5.39–7.23*   **Based on maximum dose*  **Based on budget impact models for private drug plans**  **Annual impact on budget for pain control** (average % over 3 years)**:**   - **Model 1**: +0.27% - **Model 2**: -0.32%; highly sensitive to average dose of tramadol (+2.08% for RCT doses; base case with US market doses)   ***Drugs included in models***: NSAIDs, COX-2 inhibitor, codeine, stronger opioids and Zytram; (plus meloxicam, acetaminophen in model 2); (plus slow release tramadol in model 1) | Substantial additional spending  0 1 2 3  Substantial savings  ***Comments*** |
| E2 | Cost-effectiveness of intervention | **Incremental cost (drug and adverse events) per patient over 6 months (based on cost-minimization study by Liedgens)**:   - Tramadol vs NSAIDs only: +$179 - Tramadol vs NSAIDs+ proton pump inhibitors (PPIs): -$110 (savings) - Tramadol vs NSAIDs+ histamine receprot antagonists (H2RAs): +$66   ***Adverse events included for tramadol***: constipation, nausea, vomiting, vertigo, somnolence, and others  ***Adverse events included for NSAIDs***: gastrointestinal distress, serious complications, ulcer, anemia from occult bleeding  **Sensitivity analysis**: when including renal adverse events due to NSAIDs, savings with tramadol vs NSAIDs from $345 to $631 | Not cost-effective  0 1 2 3  Highly cost-effective  ***Comments*** |
| E3 | Impact on other spending | **Impact on adverse events expenditures per patient over 6 months**: (excluding drug cost, including all adverse evnts in Liedgens study)   - Tramadol vs NSAIDs only: -$567 (savings) - Tramadol vs NSAIDs+ PPIs: -$534 (savings) - Tramadol vs NSAIDs+H2RAs: -$574 (savings)   *No data versus other comparators, no data on other spending (e.g. disability)* | Substantial additional spending  0 1 2 3  Substantial savings  ***Comments*** |
| **Quality of evidence** | | |  |
| Q2 | Completeness and consistency of reporting evidence | **Clinical data: 5 trials:** overall consistent reporting within studies but some issue in completeness of reporting including: no numerical data for efficacy (1 trial) or safety (1 trial), type of analysis unclear (1 trial), data collection schedule unclear (1 trial);  **Economic evaluation (1 cost-minimization study):** overall consistent reporting within study but some issues in completeness of reporting including: no disaggregated data regarding resource utilization and associated costs, sensitivity analyses data reported for only one parameter | Many gaps/inconsistent  0 1 2 3  Complete & consistent  ***Comments*** |
| Q3 | Relevance and validity of evidence | **Clinical data** – **5 trials:** a number of issues regarding validity and relevance including: short trial duration for chronic treatment (4 trials ≤ 6 weeks), data mostly in older osteoarthritis patients, most data for short-acting tramadol (3 trials), interpretation of results difficult (2 trials), high attrition rate (> 20% in 3 trials); efficacy measure (4–6 hrs after treatment) not relevant for chronic pain (2 trials)  **Economic evaluation (1 cost-minimization study):** a number of issues regarding validity and relevance including: Dutch setting; only NSAIDs as comparators; only adverse events considered (not consequences of these); short duration for chronic disease (6 months); only medical costs considered | Low relevance/validity  0 1 2 3  High relevance/validity  ***Comments*** |
| **Contextual Tool criteria** | | **Highly synthesized information** | **Impact on appraisal & comments** |
| **Ethical framework*** | | |  |
| Et1 | Goals of healthcare – **utility*** | **Goal of healthcare:** maintain normal functioning. Pain has a major impact on functioning and relieving pain is an ethical duty | Negative  None  Positive  ***Comments*** |
| Et2 | Opportunity costs – **efficiency*** | **Maximizing** impact on health for a given level of resources at:  **Patient level**: tramadol offers another option for pain relief but is more expensive than most other analgesics  **Population level:** Interest in using resources to treat underlying disease/condition rather than symptoms | Negative  None  Positive  ***Comments*** |
| Et3 | Population priority & access – **fairness*** | **Prioritize worst off**: Chronic pain has major ramifications for patients, family and society. Tramadol provides another option to tackle widespread undertreated chronic pain  **Treat like cases similarly**: Should chronic non-cancer pain be treated differently than cancer pain?  **Access to care/treatment**: barriers for prescribing opioids (fear of addiction and of regulatory scrutiny), critical in general practice | Negative  None  Positive  ***Comments*** |
| **Other components** | | |  |
| O1 | System capacity and appropriate use of intervention | **Risk of abuse** of opioids and difficulty to control abuse; standardized guidelines to limit abuse (mainly ensure non-opioid failed, single pharmacy/doctor and comprehensive follow-up) not always applicable in busy practices or complex cases  **Abuse rates:** lower for tramadol than for hydrocodone | Negative  None  Positive  ***Comments*** |
| O2 | Stakeholder pressures | **Canadian Pain Society**: clinician pressures on Health Canada to keep tramadol out of the controlled drug schedule on the basis that it is a good option for moderate pain and associated with less abuse than other opioids.  Currently, tramadol is not scheduled in Canada | Negative  None  Positive  ***Comments*** |
| O3 | Political /historical context | WHO committee concluded that, based on low level of abuse, there was not sufficient evidence to justify a review. WHO reports some evidence of smuggling and diversion of tramadol but no evidence of illicit manufacture;  CEDAC recommendation: do not list;  Other workplace insurance boards: reimbursement of tramadol case specific. | Negative  None  Positive  ***Comments*** |
